# Drugs Versus Microbiota: How Pharmacotherapy Affects Gut and Probiotic Bacteria

**DOI:** 10.3390/ph18091372

**Published:** 2025-09-13

**Authors:** Anna Zawistowska-Rojek, Stefan Tyski

**Affiliations:** Department of Pharmaceutical Microbiology and Laboratory Diagnostic, National Medicines Institute, 00-725 Warsaw, Poland; s.tyski@nil.gov.pl

**Keywords:** microbiota, probiotics, metformin, levothyroxine, statins, antihypertensive drugs, proton pump inhibitors

## Abstract

The gut microbiota plays a key role in digestion, nutrient absorption, immune system regulation and metabolite production, significantly impacting human health. The balance of the gut microbiota can be easily disturbed by external factors such as lifestyle, diet and drugs. Some medications—such as metformin used to treat type 2 diabetes, levothyroxine for hypothyroidism, statins for cardiovascular diseases, proton pump inhibitors for reducing stomach acid secretion, and pharmaceuticals for lowering blood pressure—can affect the balance of the gut microbiota, causing dysbiosis, which in turn may lead to other diseases. Dietary supplements, probiotics, prebiotics and certain medications alter the composition of the gut microbiota, which plays an essential role in alleviating metabolic disorders. This study discusses the effects of the long-term use of selected pharmaceuticals on the gut and probiotic microbiota in patients. It also examines whether there is a rationale for providing probiotic supplements.

## 1. Introduction

**Figure 1:**
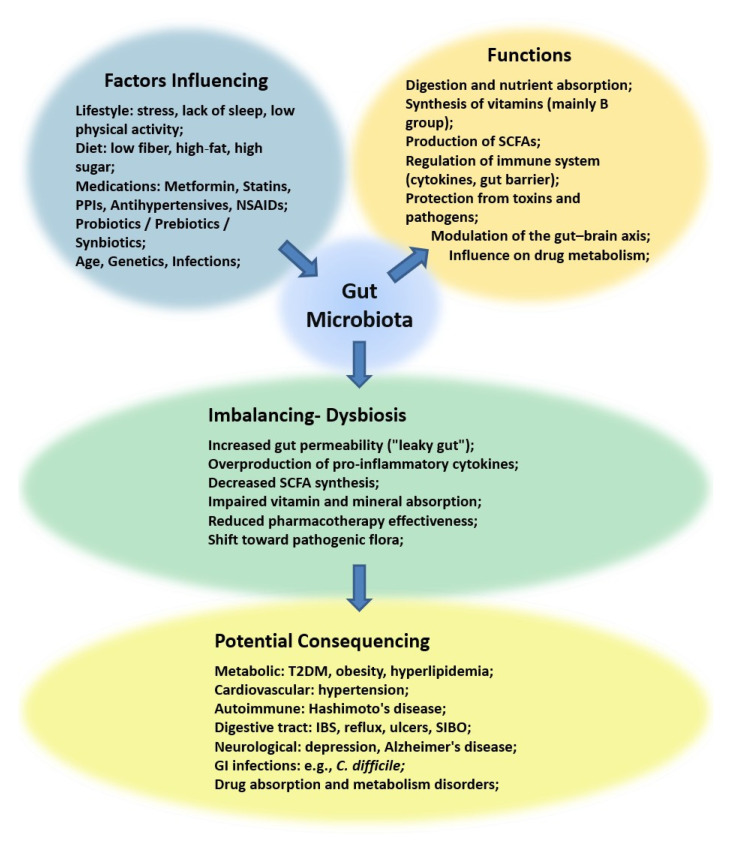
The impact of the gut microbiota on the functioning of the body. A number of different factors can affect the gut microbiota. These microorganisms are responsible for several physiological functions in humans. Disturbances in these functions can lead to microbiota imbalance—dysbiosis. This phenomenon can cause a range of diseases of a very diverse nature. SCFA—short chain fatty acids; IBS—irritable bowel syndrome; SIBO—small intestinal bacterial overgrowth; GI—gastrointestinal; PPI—proton pump inhibitor; NSAIDs—non-steroidal anti-inflammatory drugs; T2DM—type 2 diabetes mellitus.

The human microbiota is a complex ecosystem primarily inhabited by bacteria, fungi and viruses [1,2]. Numerous interactions occur among the components of this ecosystem, influencing the host’s health. The gut microbiota plays a significant role in digestion, nutrient absorption, immune system regulation and the production of certain metabolites, substantially affecting human health and disease (Figure 1) [1,3]. Microorganisms colonise the host’s body, including the skin, oral cavity, respiratory tract, and vagina, with approximately 90% of the microorganisms colonising the gastrointestinal tract. Different sections of the gastrointestinal tract are characterised by varying pH conditions, oxygen availability, nutrient levels and intestinal peristalsis, all of which influence the microorganisms colonising these different areas [2]. Aerobic microbes dominate in the small intestine, which is a more demanding environment for the microorganisms. In contrast, the large intestine, characterised by neutral to slightly acidic pH, contains the most abundant microbiota, primarily anaerobic [4]. The composition of the gut microbiota depends on the age, diet and health status of the host. The bacteria belong to four main phyla: *Firmicutes*, *Bacteroidetes*, *Proteobacteria* and *Actinomycetes*. The *Firmicutes*/*Bacteroidetes* ratio is a key parameter in assessing gut microbiota imbalances [5]. Recent research has emphasised not only these large-scale phylum-level differences but also the role of specific taxa such as *Flavonifractor plautii*, a member of the *Firmicutes* phylum, which participates in flavonoid metabolism and has been associated with host metabolic and inflammatory processes [6]. This highlights that both broad compositional shifts and the activity of particular bacterial species may contribute to health and disease. Microorganisms colonising the intestines perform several important functions (Figure 1). They participate in food digestion by secreting digestive enzymes that convert complex nutrients into simpler organic compounds. They aid in nutrient absorption and synthesise vitamins, mainly from the B group. They produce short-chain fatty acids (SCFAs), neutralise toxins and carcinogens, form a gut barrier, and have immunomodulatory functions, including cytokine regulation (Figure 1). Among SCFAs, butyric acid has emerged as one of the most crucial microbial metabolites, serving as the main energy source for colonocytes, reinforcing intestinal barrier integrity, and exerting anti-inflammatory and even anti-carcinogenic properties. Its production depends on the abundance and activity of butyrate-producing bacteria, linking microbial composition directly to host physiology [7]. Disruptions in the quantity and composition of the gut microbiota may lead to intestinal motility disorders, digestion and absorption issues, vitamin production or metabolism disorders, fat digestion difficulties, gut barrier destruction, and excessive immune system stimulation (Figure 1) [2,8]. This can result in certain illnesses, including neurodegenerative, cardiovascular, gastrointestinal and metabolic diseases (Figure 1) [5]. The balance of the gut microbiota can be easily disturbed by external factors, such as lifestyle, diet and medicines [3,9]. The most commonly described effect is the impact of antibiotics on the gut microbiota. Broad-spectrum antibiotics disrupt the balance between *Firmicutes* and *Bacteroidetes* and reduce overall bacterial diversity. The extent of these changes depends on the type of antibiotic, dosage, duration of treatment, and its mechanism of action. The antibacterial properties of antibiotics are one of the key factors driving alterations in gut microbiota composition during therapy [4]. Certain medicinal products used chronically, such as non-steroidal anti-inflammatory drugs (NSAIDs) (e.g., ibuprofen, naproxen, diclofenac, indomethacin, celecoxib) and acetylsalicylic acid, can inhibit the growth of both Gram-positive and Gram-negative bacteria, contributing to dysbiosis [9,10]. However, the maximum concentrations of these drugs in the body do not affect the survival of lactic acid bacteria [9]. An imbalance in the gut microbiota can cause serious functional disorders and diseases and also impact the effectiveness of certain medications. Metabolic diseases such as type 2 diabetes, hyperlipidaemia, hypertension, thyroid diseases, reflux and ulcers are now widespread, affecting a large part of the population. The treatments for such conditions are long-term, and patients often take medicines for the rest of their lives. The use of dietary supplements, probiotics, prebiotics and certain medicines alters the gut microbiota composition, which plays a crucial role in alleviating metabolic diseases [11]. The incidence of certain diseases, particularly metabolic disorders, has been steadily increasing in recent years, along with growing awareness of the role of gut microbiota in overall health.

The aim of this study is to investigate the impact of commonly used, chronically administered medications on the gut microbiota, with a particular focus on drugs used in the treatment of metabolic and related disorders. The work also seeks to evaluate the potential role of probiotics in supporting gut microbiota during medication intake, as well as possible interactions between medications and probiotic bacteria. Ultimately, the study aims to highlight the importance of considering drug–microbiota interactions and to explore whether and how the gut microbiota can be modulated or restored, for example, through probiotic supplementation combined with lifestyle and dietary changes.

## 2. Methodology

For the preparation of this review, a systematic literature search was conducted using the PubMed, Scopus, and Web of Science databases. The search strategy employed keywords referring both to selected chronically administered drugs and to their potential impact on the gut microbiota, as well as the rationale for concurrent probiotic supplementation. The keywords used included: *amlodipine*, *antihypertensive drugs*, *atorvastatin*, *captopril*, *hypothyroidism*, *levothyroxine*, *losartan*, *metformin*, *microbiota*, *prebiotic*, *probiotic*, *proton pump inhibitors*, *rosuvastatin*, *semaglutide*, and *statins.* Publications describing the mechanisms by which these substances affect the gut microbiota, the possible clinical consequences of such interactions, and reports addressing the efficacy and justification of probiotic use in this patient population were included.

The database search was conducted between October 2024 and August 2025 using a unified search strategy adapted to the specific requirements of each database. Only publications in English and published in peer-reviewed scientific journals were considered. Both experimental studies (in vitro, in vivo), clinical trials, and review articles were eligible for inclusion, provided that their scope addressed the effects of chronic pharmacotherapy on the gut microbiota.

Exclusion criteria encompassed anecdotal reports, single case reports without microbiological analysis, non–peer-reviewed publications (e.g., letters to the editor, commentaries), as well as articles that did not directly examine the interactions between drugs, the gut microbiota, or probiotics. Particular attention was given to studies describing the biological mechanisms underlying microbiome alterations induced by long-term pharmacotherapy, as well as those assessing the potential clinical significance of such interactions.

In addition, the reference lists of selected publications were screened to identify further relevant studies that might not have been captured through database searches. This approach ensured a more comprehensive coverage of the topic and reduced the risk of omitting valuable research. Ultimately, only studies that met strictly defined quality and thematic criteria were included, allowing for the formulation of reliable conclusions regarding the impact of chronically administered drugs on the gut microbiota and the rationale for probiotic use in this patient population.

## 3. Selected Drugs Interaction with Microbiota

### 3.1. Medications Used in the Treatment of Diabetes

Metformin is the most commonly prescribed drug for the treatment of type 2 diabetes mellitus (T2DM) [3,12,13]. T2DM is a chronic metabolic disorder characterised by hyperglycaemia and insufficient insulin secretion. The prevalence of T2DM is increasing rapidly due to changes in dietary habits, sedentary lifestyles and ageing populations. In addition to genetic and lifestyle factors, alterations in the gut microbiota also influence the development of T2DM. A diet low in fibre but high in saturated fatty acids and carbohydrates affects gut microbiota diversity, leading to metabolic dysregulation, inflammation and insulin resistance [13].

Metformin has been used for many years and is recommended by both American and European guidelines as a first-line therapy for T2DM [14]. It causes minimal risk of hypoglycaemia, has a good safety profile, has no effect on body weight and is low cost, which leads to its wide use [14]. The drug reduces gluconeogenesis, glucose absorption and glucagon-like peptide-1 (GLP-1) secretion while it increases glucose utilisation and activates adenosine monophosphate—activated protein kinase (AMPK). Beyond these classical mechanisms, metformin exerts important effects through modulation of the gut microbiota. It enhances mucin production and supports the growth of *Akkermansia muciniphila*, thereby strengthening the intestinal barrier and reducing endotoxin translocation [15]. It increases the abundance of some SCFA-producing bacteria (*Butyrivibrio*, *Bifidobacterium*, *Megasphaera*, *Prevotella*), which elevate SCFA levels that activate G-protein coupled receptors (GPR41, GPR43), stimulate GLP-1 and peptide YY secretion, improve glucose homeostasis and exert anti-inflammatory activity [15,16,17,18]. Metformin also promotes *Lactobacillus* species with bile salt hydrolase activity, altering bile acid metabolism and increasing secondary bile acids that activate FXR (Farnesoid X receptor) and TGR5 receptors (Takeda G protein-coupled receptor 5), further improving insulin sensitivity [15]. Transcriptomic studies reveal that metformin modulates microbial gene expression, including metal ion transport and metabolic pathways, while simultaneously reducing the abundance of pathogenic taxa such as *Intestinibacter* spp., *Bacteroides fragilis* and *Clostridioides difficile*, thereby lowering toxin production and inflammation [15,16,17,18]. Despite its good safety profile, metformin is associated with several gastrointestinal side effects, such as diarrhoea, abdominal pain, nausea and bloating, which affect 2–13% of patients [14], and in some reports, up to 30% of patients [19]. The exact cause of the gastrointestinal intolerance to metformin remains unclear, but hypotheses include increased lactate production, serotonin, histamine accumulation, and bile acid alterations. Metformin has been observed to influence the gut microbiota even in individuals with normal glucose metabolism, and the composition of the gut microbiota before the initiation of treatment may affect the occurrence of gastrointestinal side effects [14]. In patients with T2DM, changes in gut microbiota have been observed, including an increase in *Bacteroidetes*, *Actinobacteria*, and *Proteobacteria* with a concomitant decrease in *Firmicutes* and *Verrucomicrobia* [20]. At the genus level, metformin promotes the growth of bacteria such as *Bacteroides*, *Streptococcus*, *Collinsella*, *Escherichia*, *Clostridium* and *Subdoligranulum* while reducing the abundance of *Faecalibacterium* and *Ruminococcus* [20]. In contrast, in the study by Ermolenko et al. [17], conducted on rats with metabolic syndrome, an increase in the abundance of bacteria belonging to *Akkermansia* and a decrease in *Roseburia* bacteria were observed following metformin therapy (200 mg/kg) [17].

A meta-analysis demonstrated that the co-administration of probiotics with metformin was associated with improved glycaemic and insulin control (lower levels of fasting glucose [FG], glycated haemoglobin [HbA1c], fasting insulin [FI], and Homeostasis Model Assessment of Insulin Resistance [HOMA-IR]) compared to metformin alone. Furthermore, a reduction in gastrointestinal side effects was observed among patients who received probiotics alongside metformin [13]. In studies conducted by Sahin et al. [21], the combined use of metformin and a probiotic containing *Bifidobacterium animalis* subsp. *lactis* (BB-12) at a dose of 4.6 mg daily for one month in patients with T2DM or prediabetes led to improved glycaemic control with greater reductions in HbA1c levels compared to patients using metformin alone. The group taking probiotics also showed reduced gastrointestinal side effects, such as nausea, diarrhoea, abdominal pain and bloating. Clinical trials by Hata et al. [22] using the probiotic strain *Bifidobacterium bifidum* G9-1 (BBG9-1) at a dose of 216 mg daily for 10 weeks during metformin treatment of T2DM patients demonstrated significant improvements in gastrointestinal symptom scores (e.g., constipation and diarrhoea) following probiotic administration, with no change in glycaemic control. In studies by Nabrdalik et al. [14], patients with T2DM taking metformin at doses not exceeding 1500 mg per day were supplemented with the multi-strain probiotic Sanprobi Barrier, containing strains such as *B. bifidum* W23, *B. lactis* W51 and W52, *Lactobacillus acidophilus* W37, *Levilactobacillus brevis* W63, *Lacticaseibacillus casei* W56, *Ligilactobacillus salivarius* W24, *Lactococcus lactis* W19 and W58 at a dose of 2 × 10^9^ CFU/day. The administration of this multi-strain probiotic was associated with alleviation in the gastrointestinal side effects in metformin-treated patients. In the group receiving the probiotic alongside metformin, the frequency of the side effects decreased significantly [14].

It is also worth noting that an improvement in gut microbiota composition with metformin use has been observed when combined with prebiotics. In a randomised clinical trial conducted in a group of adolescents with obesity, who received metformin (1000 mg/day) together with the prebiotic—oligofructose (8 g/day), beneficial changes in the gut microbiota were observed compared to the group receiving metformin alone. An increased abundance of SCFA-producing bacteria, such as *Bifidobacterium adolescentis*, *Blautia*, and *Acinetobacter*, was reported, along with a reduction in other bacterial groups, including *Firmicutes*, *Roseburia*, and *Enterobacteriaceae* [23].

Semaglutide has gained popularity for the management of T2DM in recent years,. Approved by the Food and Drug Administration (FDA) in 2017 and European Medicines Agency (EMA) in 2018, semaglutide is a GLP-1 receptor agonist [24]. It has shown efficacy in weight reduction, improved glycaemic control, and decreased cardiometabolic risk factors in T2DM patients [25]. Research on its impact on the gut microbiota remains limited. Some researchers on animal models were conducted, and their findings provide evidence of semaglutide’s impact on gut microbiota. Studies by Mao et al. [26] in db/db mice observed alterations in the gut microbiota after semaglutide administration (0.22 mg/kg once every 3 days), including an increased abundance of *Alloprevotella* and *Alistipes* and reduced populations of *Ligilactobacillus* and *Lactobacillus*. Hu et al. [27] reported that semaglutide treatment (30 nmol/kg per day) increased the abundance of *Lactobacillus* and *Limosilactobacillus* in mice. Feng et al. [25] found that a 12-week semaglutide treatment (30 nmol/kg per day) in mice increased the populations of *Akkermansia*, *Muribaculaceae*, *Coriobacteriaceae* UCG-002 and *Clostridia* UCG-014 while reducing the abundance of *Romboutsia*, *Dubosiella* and *Enterorhabdus*. These findings support the hypothesis that semaglutide may help regulate gut microbiota imbalances caused by high-fat diets [25].

### 3.2. Medications Used in the Treatment of Hypothyroidism

Levothyroxine is a medicine used to treat hypothyroidism, which is often associated with autoimmune thyroiditis, also known as Hashimoto’s disease, which affects approximately 5% of the population [28,29], predominantly women [30]. Hypothyroidism can impair gastrointestinal motility, potentially leading to dyspepsia, irritable bowel syndrome (IBS) and small intestinal bacterial overgrowth (SIBO) [30]. It may also be linked to cardiovascular diseases such as hypertension, dyslipidaemia and depression [31]. A lower *Firmicutes*/*Bacteroidetes* ratio alongside increased pathogenic bacteria and decreased populations of *Lactobacillus* and *Bifidobacterium* have been observed in individuals with autoimmune thyroiditis compared to healthy individuals [32]. The microbiome also influences thyroid hormones. Bacterial thyroid-binding protein (bTBP) binds triiodothyronine (T3) and thyroxine (T4), which can affect thyroid hormone fluctuations and necessitates modifications in levothyroxine dosage [33]. Levothyroxine is the standard treatment for hypothyroidism [34]. An overgrowth of bacteria can disrupt the absorption of oral thyroxine [31]. In addition, the gut microbiota regulates the absorption of trace elements essential for thyroid hormone synthesis, such as iodine, iron, selenium, zinc and copper. The microbiota can also produce inflammatory cytokines, which are associated with hypothyroidism [35]. At the molecular level, gut bacteria are able to bind T4 and T3 molecules directly, acting as a reservoir of thyroid hormones and competing with host proteins for binding. Moreover, bacterial β-glucuronidase deconjugates glucuronidated or sulphated forms of T4 in the intestine, releasing active hormone and altering its enterohepatic recirculation. These mechanisms directly influence levothyroxine bioavailability [36]. On a cellular level, levothyroxine–microbiota interactions impact intestinal barrier integrity by modifying the expression of tight junction proteins, mucus layer composition and enterocyte morphology. This affects intestinal permeability and the efficiency of levothyroxine absorption [37]. Through these interactions, levothyroxine also indirectly influences bacterial metabolites such as SCFAs and secondary bile acids. These metabolites regulate immune responses, strengthen epithelial integrity, and activate pathways such as FXR/TGR5 signalling and type 2 deiodinase, linking levothyroxine therapy to systemic metabolic and immune effects via the gut microbiota [36].

In clinical studies, an effect of the administered dose of levothyroxine in patients with subclinical hypothyroidism on the composition of the gut microbiota was observed. With increasing drug dosage, the abundance of bacteria belonging to the genera *Odoribacter* and *Enterococcus* increased. A statistically significant difference was also observed in the abundance of the genera *Alistipes*, *Ruminococcus*, and *Anaerotruncus* between the group of patients whose dose was increased during the study and the group of patients with a stable dose [38].

There is growing interest in exploring the role of probiotic supplementation in improving thyroid function. In studies conducted by Talebi et al. [31], the administration of a synbiotic containing seven bacterial strains (*L. casei*, *L. acidophilus*, *Lacticaseibacillus rhamnosus*, *Lactobacillus bulgaricus*, *B. breve*, *B. longum* and *Streptococcus thermophilus*) at a dose of 7 × 10^9^ CFU/capsule along with fructooligosaccharides for eight weeks resulted in reductions in thyroid-stimulating hormone (TSH), free triiodothyronine (FT3) and levothyroxine dosage. However, the observed outcomes were not statistically significant. In another study by Spaggiari et al. [34], patients were administered a probiotic labelled as VSL#3, comprising a mixture of *B. breve*, *B. longum*, *Bifidobacterium infantis*, *L. acidophilus*, *Lactiplantibacillus plantarum*, *L. casei*, *L. bulgaricus* and *S. thermophilus*, at a dose of 4.5 × 10^11^ CFU/sachet for four months. No significant differences in parameters between the intervention and control groups were noted. However, it was hypothesised that levothyroxine treatment would be stabilised after probiotic consumption, as suggested by the reduced hormone dosage required in the probiotic group [34]. In randomised double-blind clinical trials, patients with hypothyroidism treated with levothyroxine received a synbiotic containing *L. casei*, *L. acidophilus*, *L. rhamnosus*, *L. bulgaricus*, *B. breve*, *B. longum*, *S. thermophilus* (10^9^ CFU/g) and fructo-oligosaccharide. Ten-week supplementation did not significantly improve TSH and FT4 (free thyroxine) levels; however, an improvement in blood pressure and quality of life was observed in the studied patients [35].

Hao et al. [39] investigated pregnant women with hypothyroidism, some of whom also had SIBO. A probiotic containing *Bifidobacterium*, *L. acidophilus*, *Enterococcus faecalis* and *Bacillus cereus* was administered at a dose of 1.5 g three times daily for 21 days. Normalisation of thyroid hormone and TSH levels was observed in patients with SIBO after 21 days of therapy. Probiotic and prebiotic use can enhance intestinal mucosal barrier integrity, stabilise TSH levels and improve thyroid function [39].

### 3.3. Medications Used to Lower Cholesterol Level

Statins are medicines widely used in the treatment of cardiovascular diseases. Their mechanism of action is based on reducing the levels of low-density lipoprotein-cholesterol (LDL-C), commonly referred to as “bad cholesterol”. They work by inhibiting 3-hydroxy-3-methylglutaryl-coenzyme A (HMG-CoA), thereby decreasing de novo cholesterol synthesis within the body [40,41]. Statins lower the risk of cardiovascular diseases, reduce the production of pro-inflammatory cytokines in cells, and exhibit antioxidant properties [40]. They also have direct antibacterial effects, can act synergistically with antibiotics (e.g., the synergistic effect of simvastatin with daptomycin, fusidic acid, mupirocin and retapamulin against *Staphylococcus aureus*), and stimulate the host immune system [40,42]. Studies conducted on large populations suggest that statins might be associated with the risk of T2DM, depending on the dosage administered [43]. The long-term use of such medicines may impact gut microbiota diversity, potentially leading to dysbiosis [42]. In addition, reduced risks of *Clostridioides difficile* infections and colorectal cancer have been observed in patients with inflammatory bowel disease [44]. Several factors, such as physical activity, smoking habits, dietary choices, nutraceuticals [45] and genetic predispositions [46], may influence blood lipid levels and the efficacy of statins. A high-fat diet significantly affects the composition of the gut microbiota, directly contributing to obesity, insulin resistance and other metabolic disorders. Among obese individuals, an increased prevalence of bacteria belonging to the *Firmicutes* phylum and a reduced prevalence of bacteria from the *Bacteroidetes* phylum have been observed. Further research showed that the gut microbiota may influence cholesterol metabolism and interact with cholesterol-lowering drugs such as pravastatin and simvastatin [47]. The most commonly used statins include rosuvastatin and atorvastatin, which share similar pharmacokinetics, efficacy and side effects—potentially attributed to their comparable effects on the microbiota. However, differences in gut bacterial composition have been observed following the administration of rosuvastatin and atorvastatin, which may stem from disparities in their chemical structure and influence on hyperlipidaemia and glucose metabolism. While atorvastatin is lipophilic, rosuvastatin is hydrophilic and demonstrates greater efficacy in lowering cholesterol levels. It remains unclear how these differences affect the gut microbiota [48]. At the molecular level, statins influence bile acid metabolism by reducing hepatic cholesterol synthesis and thereby remodelling the bile acid pool. This shift alters the abundance of bacteria capable of metabolising bile acids and modulates farnesoid X receptor (FXR) and cell surface receptor G protein-coupled bile receptor 5 (TGR5) signalling, which are crucial for host lipid and glucose regulation [49,50]. On the cellular level, statins induce changes in microbiota composition, including an increase in beneficial taxa such as *Akkermansia muciniphila* and *Lactobacillus*, alongside a decrease in pro-inflammatory bacteria. These changes support intestinal barrier integrity and regulate tight junction proteins, directly affecting gut permeability [41,50]. Furthermore, statins shape the profile of bacterial metabolites, including SCFAs and secondary bile acids. These metabolites act as signalling molecules that regulate host gene expression involved in lipid metabolism, immune modulation, and inflammatory pathways [41,49]. Lipophilic statins, such as simvastatin, also exhibit direct antibacterial activity by disrupting bacterial cell membranes and interfering with bacterial mevalonate pathways, acting as a selective force within the gut ecosystem [50]. Finally, statin-induced microbiota remodelling attenuates LPS–TLR4 signalling and reduces low-grade systemic inflammation, which is strongly linked to the development of atherosclerosis [41,49].

Certain probiotic strains, such as *L. acidophilus*, *L. plantarum*, *L. casei*, *B. longum*, *E.faecalis* and *S. thermophilus*, influence the reduction in total cholesterol and LDL-C levels in the bloodstream [51]. Studies by Wang et al. [52] demonstrated that administering the probiotic strain *L. acidophilus* ATCC 4356 to rats with high cholesterol for four weeks resulted in reductions in total cholesterol of 20.1% and LDL-C of 39.7% compared to the control group, indicating the effective cholesterol-lowering potential of *L. acidophilus*.

Rosuvastatin is a third-generation statin used in coronary heart disease prevention. In addition to reducing total cholesterol, it influences other lipid profiles, including triglycerides (TG). Therapy with statins has proved insufficient for about 20% of patients, irrespective of increased dosages. It is notable that high doses of statins are prone to triggering adverse effects, including muscle pain, weakness and liver damage [46,52]. A connection between the gut microbiota and hyperlipidaemia has been observed, as individuals with hyperlipidaemia often exhibit an altered gut microbiota composition, characterised by increased numbers of Gram-negative bacteria and reduced numbers of SCFA-producing bacteria [46]. Research by Wang et al. [45] revealed that the gut microbiota affects the action of rosuvastatin (10 mg/kg). Rats administered antibiotics with statins showed decreased gut microbiota diversity and reduced abundance of *Lactobacillus* and *Bifidobacterium*, leading to significantly higher LDL-C and TG levels compared to those receiving statins alone. After restoring the gut microbiota, rosuvastatin’s lipid-lowering effects returned to normal levels. Moreover, a synergistic effect between *L. acidophilus* and rosuvastatin (10 mg/kg) was observed in the studied rats. After two weeks of therapy, total cholesterol levels were reduced by 25.6% and LDL-C levels by 39.7% [52]. In addition, the population of *L. acidophilus* isolated from faecal samples increased in the groups under analysis. The randomised controlled trial conducted by Kummen et al. [44] on a group of patients did not show any changes in bacterial taxonomic groups in individuals treated with rosuvastatin (20 mg/day). In turn, in a study conducted on mice by Kim et al. [11], administration of rosuvastatin at a dose of 3 mg/kg body weight was associated with an increased relative abundance of *Bacteroides*, *Butyricimonas*, *Clostridium*, and *Mucispirillum*. Moreover, a reduction in the *Firmicutes/Bacteroidetes* ratio was observed, which paralleled the metabolic improvements elicited by statin treatment. Liu et al. [46] identified different gut microbiota compositions in patients on rosuvastatin (10 mg/day). Patients with greater faecal microbiome complexity, characterised by higher abundances of bacteria from the *Firmicutes* phylum and families *Ruminococcaceae*, *Lachnospiraceae* and *Clostridiaceae*, as well as lower counts of *Bacteroidetes* bacteria, experienced optimal therapeutic effects from rosuvastatin. *Firmicutes* participate in the metabolism of phenolic compounds, which act as antidiabetic and anti-obesity agents, potentially playing a role in maintaining normal blood lipid levels. Families such as *Ruminococcaceae*, *Lachnospiraceae* and *Clostridiaceae* ferment dietary fibre into SCFAs, such as butyrate, which reduce the occurrence of liver steatosis, inflammatory bowel disease and diabetes [46].

Atorvastatin is another commonly used statin. This compound partially reverses the gut microbiome alterations caused by a high-fat diet. However, its lipid-lowering efficacy is significantly weakened in cases of an impoverished gut microbiome. Furthermore, the depletion of the gut microbiome substantially changes atorvastatin’s effects on various liver and intestinal genes involved in cholesterol metabolism [53]. Moreover, atorvastatin may impair glucose tolerance and promote the development of diabetes by mechanisms such as increasing hepatic gluconeogenesis or delaying glucose clearance [43]. In a study conducted on mice by Cheng et al. [43], a decrease in the *Bacteroides/Firmicutes* ratio was observed in faecal samples from animals administered atorvastatin at a dose of 10 mg/kg body weight per day. Moreover, an increased abundance of *Oscillibacter*, *Turicibacter*, *Anaerovorax*, and *Parvibacter* was noted, along with a decreased abundance of *Parabacteroides*, *Akkermansia*, *Rikenella*, and *Christensensellaceae*. The authors suggested that the dysregulated glycaemic levels associated with atorvastatin use may be linked to its impact on the gut, including changes in microbiota composition, impaired gut barrier function and subsequent chronic inflammation. In a study conducted on mice by Kim et al. [11], administration of atorvastatin at a dose of 10 mg/kg body weight resulted in an increased relative abundance of *Anaerotruncus*, *Bacteroides*, *Butyricimonas*, *Dorea*, *Mucispirillum*, and *Turicibacter*. A reduction in the *Firmicutes/Bacteroidetes* ratio was also observed. In turn, in a study conducted in humans, administration of atorvastatin at a dose of 20 mg/day was associated with a significant reduction in the abundance of *Bifidobacterium*, *Tyzzerella*, and *Lactobacillus* compared with the DMSO group [54].

In randomised, double-blind, placebo-controlled clinical trial by Tian et al. [55], a group of patients was administered 2 g of probiotics daily (*L. casei* Zhang, *B. animalis* subsp. *lactis* V9 and *L. plantarum* P-8) together with atorvastatin (20 mg). Significant reductions in total cholesterol, TG and LDL-C were noted in the group taking probiotics with atorvastatin compared to the control group receiving a placebo with atorvastatin at the same dose. The probiotic-supplemented group also exhibited greater gut microbiota diversity, with increased populations of *Tenericutes*, *Bifidobacterium*, *Lactobacillus* and *Akkermansia* and decreased populations of *Proteobacteria*, *Escherichia*, *Eggerthella* and *Sutterella* [55].

### 3.4. Medications Used to Lower Blood Pressure

Hypertension is recognised worldwide as a risk factor for cardiovascular diseases [56]. According to data from the World Heart Federation [57], hypertension affects as many as 1.3 billion people and accounts for approximately half of all deaths caused by heart diseases and strokes. Hypertension does not produce symptoms, which is why it is often referred to as the “silent killer” [57]. Researchers hypothesise [56] that hypertension results from individual genetic factors and an unhealthy lifestyle. The widespread use of antihypertensive drugs has led to a global decrease in average blood pressure. However, over the past few decades, the problem of hypertension has resurged due to ageing populations and exposure to risk factors such as high sodium intake, low potassium intake, alcohol consumption, physical inactivity, unhealthy diets, smoking, air pollution, stress, noise and the lack of sleep [57]. Recent studies have suggested a relationship between the gut microbiota and hypertension and also cardiovascular diseases [56,58,59,60]. Patients with hypertension have been found to exhibit gut microbiota imbalances, altered microbial metabolite production, and pathological changes in the gut [56]. Increased populations of bacteria from genera such as *Klebsiella*, *Parabacteroides*, *Salmonella*, *Prevotella*, *Enterobacter*, *Alistipes*, *Streptococcus*, *Eggerthella*, *Fusobacterium*, *Anaerotruncus*, *Ruminococcus* and *Eubacterium* were observed, along with reduced numbers of bacteria from genera such as *Faecalibacterium*, *Synergistetes*, *Bacteroidetes*, *Oscillibacter*, *Butyrivibrio*, *Paraprevotella*, *Roseburia*, *Bifidobacterium* and *Coprococcus* [48,61,62]. Studies by Dinakis et al. [63] revealed that certain bacterial taxa are directly associated with blood pressure variability. Increased numbers of *Lactobacillus* and *Alistipes finegoldii* bacteria were observed in individuals with normal blood pressure, whereas higher numbers of *Clostridium* and *Prevotella* spp. bacteria were found in individuals with elevated blood pressure [63]. Research by Palmu et al. [64] involving a group of 6953 Finnish participants identified correlations between blood pressure and the presence of 45 microbial genera, 27 of which belong to *Firmicutes*. In addition, negative associations were found between 19 species of *Lactobacillus* and blood pressure indices [64]. Studies have also demonstrated that the gut microbiota influences the therapeutic effects of antihypertensive drugs, affecting their absorption, metabolism and pharmacokinetics [56]. Research involving human and rat faecal suspensions showed that the gut microbiota reduced the bioavailability of amlodipine, a calcium channel blocker, by 21.3% within 72 h [65]. Importantly, recent pharmacomicrobiomic research indicates that these interactions are bidirectional: gut bacteria metabolise drugs through enzymatic reactions (e.g., esterases, reductases, oxidases, deacetylases), which can reduce their bioavailability, while antihypertensive drugs in turn reshape the gut environment, altering bile acid composition, luminal pH, and key metabolites such as SCFAs and trimethylamine N-oxide (TMAO) [66,67]. It is also important to highlight that despite the wide variety of antihypertensive drugs available, approximately 10–20% of patients are not susceptible to treatment [59,68]. This means that their blood pressure remains elevated despite the administration of three different types of antihypertensive medicines, including one diuretic [59].

There are 23 classes of antihypertensive drugs, encompassing over 100 different medicines that operate through various mechanisms. The most commonly used include calcium channel blockers, angiotensin-converting enzyme inhibitors (ACEi), angiotensin II receptor antagonists, β-blockers and thiazide diuretics [60,66,69]. Frequently prescribed medicines include amlodipine, captopril, losartan and benazepril [60].

Amlodipine, used widely in hypertension treatment, functions by blocking calcium channels. It is well absorbed from the gastrointestinal tract, with an oral bioavailability of approximately 60% [65]. In vitro studies by Kruszewska et al. [70] demonstrated that amlodipine exhibited inhibitory effects against pathogenic bacteria (MIC 0.1–0.2 mg/mL), fungi (MIC 0.1 mg/mL), and bacteria from the *Lactobacillaceae* genus (MIC 0.1–0.2 mg/mL). Studies by Yoo et al. [65] conducted on rats indicated that amlodipine’s bioavailability can be enhanced through the use of antibiotics, which inhibit the metabolic activity of the gut microbiota. Li et al. [71] observed the effects of amlodipine aspartate (2.8 mg/kg and 1.4 mg/kg per day) and amlodipine besylate (2.8 mg/kg and 1.4 mg/kg per day) on gut dysbiosis in mice, noting increased populations of *Akkermansia*, *Bacteroides* and *Lactobacillus*. In mice, the administration of amlodipine besylate resulted in increased numbers of *Firmicutes* bacteria, particularly *Ruminoclostridium* and *Lachnospiraceae*, while amlodipine aspartate led to increased *Bacteroidetes* and *Verrucomicrobia* populations [71]. Moreover, Saputri et al. [72] administered the probiotic strain *L. plantarum* IS-10506 to rabbits, observing significantly higher plasma concentrations of unmetabolised amlodipine in the probiotic-treated group. The authors suggested two mechanisms for the increased absorption of unmetabolised and pharmacogenetically active amlodipine: enhanced oxygen delivery and blood flow, which promote drug absorption, and increased plasma protein concentrations, which improve amlodipine binding and uptake [72]. At the molecular level, calcium channel blockers such as amlodipine are also subject to oxidative and reductive transformations by microbial enzymes, while at the community level they shift bacterial composition, often increasing *Firmicutes* and reducing *Bacteroidetes* [66,67].

Captopril, an ACEi drug, lowers blood pressure by suppressing the rein-angiotensin system [66,73]. Studies conducted by Yang et al. [73] in rats demonstrated that captopril (250 mg/kg/day) affects gut microbiota composition, intestinal permeability, pathology and brain activity in the posterior pituitary lobe, even after discontinuing the drug. Long-term effects of captopril were observed in bacterial populations such as *Parabacteroides*, *Mucispirillum* and *Allobaculum*. Compared to the control group of rats, those treated with captopril showed increased populations of *Tenericutes*, *Actinobacteria*, *Proteobacteria* and *Firmicutes* and decreased *Bacteroides* populations [73]. In addition, both captopril and amlodipine increased intestinal leakage and boosted populations of acetate-producing anaerobic bacteria [74]. Captopril also influences intestinal integrity by modulating the expression of tight junction proteins (e.g., ZO-1, occludin), thereby altering epithelial permeability, and through suppression of excessive sympathetic signalling it helps restore beneficial taxa such as *Lactobacillus* and *Bifidobacterium* [66,67].

Losartan, classified as an angiotensin II receptor antagonist, is among the most commonly used antihypertensive drugs, with its actions closely tied to the gut microbiota [66]. Robles-Vera et al. [75] examined losartan’s effects on the gut microbiota in spontaneously hypertensive rats. Losartan treatment (20 mg/kg/day) was shown to reduce gut dysbiosis, increase the abundance and diversity of the gut microbiota, restore the appropriate *Firmicutes/Bacteroidetes* ratio, and elevate populations of anaerobic bacteria. Specific changes included increased populations of families such as *Verrucomicrobiaceae*, *Pedobacter* and *Akkermansia*, along with reduced populations of *Lactobacillaceae* and *Lactobacillus*. These alterations were independent of losartan’s blood-pressure-lowering effects but correlated with improved intestinal integrity and normalised production of α-defensins in the colon [75]. Consistently, other studies confirm that losartan strengthens the epithelial barrier by upregulating tight junction proteins, supports the growth of *Akkermansia muciniphila*, and modifies bile acid and metabolite profiles, highlighting a dual molecular and cellular mechanism of action on the microbiota [66,67,76].

In addition to conventional pharmacotherapy aimed at reducing blood pressure, probiotic microorganisms have been shown to lower blood pressure in both humans and animals. Modulating the gut microbiota and its metabolites could serve as a preventive measure against hypertension. The findings from nine randomised controlled trials indicated reductions of 3.56 mmHg in systolic blood pressure and 2.38 mmHg in diastolic blood pressure in patients receiving probiotics. Greater efficacy in lowering blood pressure was observed when multiple probiotics were administered simultaneously, with effectiveness depending on the strain and dosage used [77]. In randomised studies involving 30 patients diagnosed with hypertension, the daily administration of probiotic strains *Lactobacillus helveticus* (7.0 × 10^11^ CFU/day) and *Saccharomyces cerevisiae* (2.5 × 10^9^ CFU/day) for 8 weeks led to reductions in systolic blood pressure of 14.1 mmHg and diastolic blood pressure of 6.9 mmHg [78]. Similarly, research on 70 individuals with normal blood pressure values showed that consuming yoghurt containing *E. faecium* and *S. thermophilus* for 8 weeks reduced systolic blood pressure by 8 mmHg and diastolic blood pressure by 4 mmHg [79]. Naruszewicz et al. [80] conducted studies with 36 healthy individuals who smoked, administering a drink containing the probiotic strain *L. plantarum* 299v (5 × 10^7^ CFU/day) over a 6-week period. Reductions in systolic blood pressure of 13 mmHg and diastolic blood pressure of 5 mmHg were observed

### 3.5. Medications Used to Reduce Gastric Acid Secretion

Proton pump inhibitors (PPIs) are medicines that reduce gastric acid secretion. This group includes omeprazole, esomeprazole, lansoprazole, dexlansoprazole, pantoprazole and rabeprazole [81]. Their action begins in the stomach’s acidic environment, where they become activated and inhibit the proton-potassium pumps (H^+^/K^+^ ATPases), membrane proteins responsible for releasing hydrochloric acid into the stomach lumen [82,83]. PPIs are commonly used to manage gastrointestinal disorders, such as stomach ulcers, erosive oesophagitis and gastro-oesophageal reflux disease (GORD) [82,83]. At the molecular level, PPIs act as prodrugs that, once protonated in the acidic canaliculi of gastric parietal cells, are converted into active sulphonamides which covalently bind to cysteine residues of the H^+^/K^+^-ATPase, leading to an irreversible inhibition of acid secretion and elevation of gastric pH [81]. They are also employed prophylactically to prevent peptic ulcers, particularly during the use of certain medicines such as NSAIDs, aspirin and steroids [83]. In addition, PPIs are administered alongside antibiotics to eradicate *Helicobacter pylori* infections [81]. PPIs rank among the most widely used drugs globally [84]. However, it is estimated that 25–70% of prescriptions for these medicines may be inappropriate, with patients continuing to use them without sufficient evidence supporting their necessity [83,85,86]. Although generally considered safe, common side effects of PPIs include headaches, diarrhoea, abdominal pain, nausea, vomiting and bloating, occurring in about 5% of patients [85]. Other potential risks include pneumonia and neurodegenerative conditions (e.g., dementia) due to the effects on the gut–brain axis [81]. Long-term PPI use may also increase the likelihood of gastrointestinal infections (e.g., *C. difficile*), nutritional deficiencies, and bone fractures potentially linked to the impaired absorption of calcium, vitamin B12 and iron, as well as dysbiosis [81,83,85,86,87]. PPIs impact the gut microbiota through their effects on stomach acid. By suppressing acid secretion, they weaken the antimicrobial barrier of the stomach, which allows oral bacteria such as *Streptococcus* and *Rothia* to survive and translocate into the gut, a process described as oral–gut microbial translocation [88]. Lowering gastric acidity allows more bacteria to overcome the stomach barrier and reach the intestines [81,84]. An increase in gastric bacterial populations was observed in healthy individuals taking omeprazole daily for two weeks [84]. Furthermore, after PPI use, oral-cavity bacteria were found throughout the upper and lower gastrointestinal tracts [84]. Beyond this indirect effect, PPIs may also directly inhibit the growth of selected commensals while promoting opportunistic species, thereby shifting the balance of the gut microbiota [88]. Hypochlorhydria has additionally been shown to reduce beneficial taxa such as *Faecalibacterium*, which produce anti-inflammatory SCFAs, thus enhancing susceptibility to inflammation [81]. Moreover, PPIs interfere with host immunity by blocking vacuolar H^+^-ATPases in neutrophils, impairing phagolysosomal acidification and oxidative burst, and suppressing inhibitory factor κB-α (IκB-α), extracellular signal-regulated kinase (ERK) and Janus kinase/ Signal transducer and activator of transcription (JAK/STAT) signalling, leading to reduced production of pro-inflammatory cytokines (e.g., tumour necrosis factor alpha—TNF-α, Interleukin 1 beta—IL-1β, Interleukin 8—IL-8) [81]. These molecular effects contribute synergistically to dysbiosis, increased intestinal permeability, and microbial translocation of bacterial products such as lipopolysaccharide (LPS), which can trigger systemic immune activation [89]. The use of PPIs has also been associated with a higher prevalence of SIBO, likely stemming from the loss of the stomach acid’s defensive barrier. Jejunal samples from SIBO patients showed overgrowth of microaerophilic microorganisms such as *Streptococcus*, *Staphylococcus*, *Escherichia* and *Klebsiella* and anaerobes such as *Bacteroides*, *Lactobacillus*, *Veillonella* and *Clostridium* [89,90].

Dysbiosis is frequently reported among individuals using PPIs, and is characterised by a loss of microbial diversity, oralisation, and bacterial overgrowth in the small intestine. Changes in the microbiota composition include reduced populations of *Clostridiales* and increased populations of *Enterococcaceae* and *Streptococcaceae*, which may predispose patients to *C. difficile* infections [87]. Amir et al. [91] observed alterations in the oesophageal and gastric microbiota diversity before and after administering lansoprazole at 30 mg twice daily for 8 weeks. In oesophageal biopsies, decreases in *Comamonadaceae* and increases in bacteria from groups such as *Clostridia* (*Clostridiaceae*, *Lachnospiraceae*), *Actinomycetales* (*Micrococaceae*, *Actinomycetaceae*), *Lactobacillales* and *Gemellales* were noted. Gastric fluid analysis revealed decreased populations of *Moraxellaceae*, *Flavobacteriaceae*, *Comamonadaceae* and *Methylobacteriaceae*, alongside increased *Erysipelotrichaceae* and *Clostridiales* [91]. Other researchers have described extensive changes in the microbiota across different sections of the gastrointestinal tract due to PPI use [89]. For example, studies have noted increases in *Fusobacteriaceae* and *Leptotrichiaceae* in the oral cavity, *Micrococaceae*, *Actinomycetaceae* and *Clostridiaceae* in the oesophagus, *Streptococcaceae* in the stomach, *Streptococcaceae*, *Staphylococcaceae*, *Enterobacteriaceae*, *Bacteroidaceae*, *Lactobacillaceae*, *Veillonellaceae* and *Clostridiaceae* in the small intestine, and *Enterobacteriaceae*, *Enterococcaceae* and *Lactobacillaceae* in the colon. Conversely, decreases were observed in *Neisseriaceae* and *Veillonellaceae* in the oral cavity, *Comamonadaceae* in the oesophagus, *Prevotellaceae* in the stomach, *Bifidobacteriaceae* in the small intestine, and *Ruminococcaceae* and *Bifidobacteriaceae* in the colon [89]. A meta-analysis also reported increased populations of *Bifidobacterium dentium* in the intestines of individuals using PPIs [92]. In randomised clinical trials, administration of esomeprazole at a dose of 40 mg/day was observed to increase the abundance of *Streptococcus* in the gut microbiota, originating from the oral cavity or the oral/nasal sites [86]. In studies by Hojo et al. [90], patients who took PPIs including esomeprazole (20 mg), rabeprazole (10 mg) and lansoprazole (30 mg) daily for 8 weeks exhibited increased populations of *Lactobacillus gasseri*, *L. reuteri*, *Limosilactobacillus fermentum*, *Ligilactobacillus ruminis*, *L. brevis*, *Streptococcus* species and facultative anaerobes such as *Staphylococcus* and members of *Enterobacteriaceae*. Administering probiotic strains has shown potential in reducing SIBO associated with PPI use. In a study by Del Piano et al. [93], patients taking PPIs were given probiotics containing *L. rhamnosus* LR06 (DSM 21981), *Lactiplantibacillus pentosus* LPS01 (DSM 21980), *L. plantarum* LP 01 (LMG P-21021) and *L. delbrueckii* subsp. *delbrueckii* LDD01 (DSM 22106) for 10 days at a dosage of 1 × 10^10^ CFU/sachet. A significant decrease in populations of enterococci, coliform bacteria, *E. coli*, moulds and yeasts was observed compared to the non-probiotic group. In another study, children with GORD taking PPIs (esomeprazole, 1 mg/kg daily) for 12 weeks received the probiotic strain *L. reuteri* DSM 17938 at 1 × 10^8^ CFU/day. Dysbiosis, observed in 56.2% of the placebo group, was reduced to 6.2% in the probiotic-treated group. A reduction in bacterial overgrowth was also noted [94]. Horvath et al. [87] provided patients on PPIs for more than six months a probiotic product containing 12 bacterial strains, including *Bacillus coagulans*, *B. subtilis*, *B. bifidum*, *B. lactis*, *L. acidophilus*, *L. casei*, *L. rhamnosus*, *L. salivarius*, *L. lactis* and *Propionibacterium freudenreichii* at 2 × 10^9^ CFU/g, in a daily dose of 4 g. After three months, improvements in intestinal permeability and liver biochemical parameters were noted, though inflammation and microbiota composition remained unchanged. Kwak et al. [95] administered probiotics (*B. bifidum*, *B. lactis*, *B. longum*, *L. acidophilus*, *L. rhamnosus* and *S. thermophilus*) at 5 × 10^9^ CFU/capsule twice daily to patients with chronic liver disease. After four weeks, changes in faecal bacteria composition and alleviation of SIBO symptoms were observed, but no effects on intestinal permeability or liver function were recorded. Singh et al. [96] conducted a double-blind randomised clinical trial where two groups of patients received omeprazole (20 mg/day) or placebo. After two weeks, increased populations of *Streptococcaceae* were observed in the PPI group. Both groups were then given the VSL probiotic containing eight strains (e.g., *S*. *thermophilus*, *B. breve*, *L. acidophilus*, *L. plantarum*) at 9 × 10^11^ CFU/day. After four weeks, the PPI group showed significant increases in probiotic bacteria such as *L. acidophilus*, *L. plantarum*, *S. thermophilus* and *B. animalis*. The placebo group displayed increases in only three strains (*B. animalis*, *L. plantarum* and *L. acidophilus*). The study suggested that PPIs facilitate colonisation by certain probiotic bacteria, such as *S. thermophilus* [96].

### 3.6. Summary

**Figure 2:**
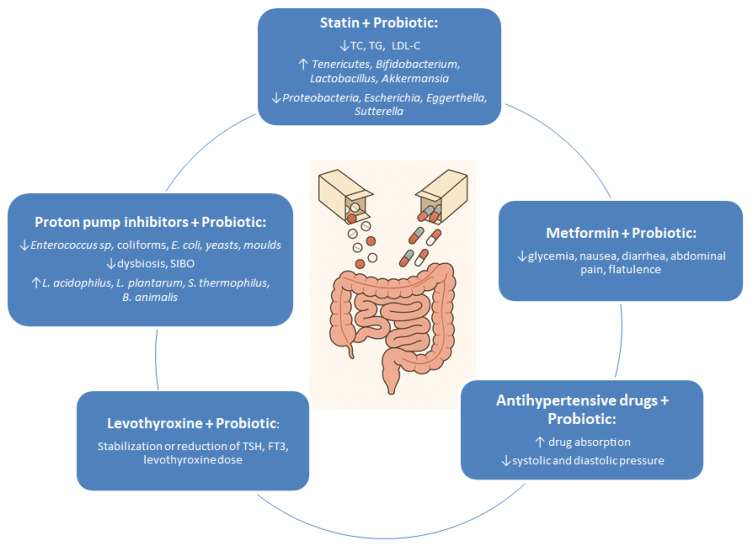
The impact of interactions between certain medicines and probiotics on the gut. TC—total cholesterol; TG—triglycerides; LDL-C—low-density lipoprotein-cholesterol; SIBO—small intestinal bacterial overgrowth; TSH—thyroid-stimulating hormone; FT3—free triiodothyronine; ↑—increase level; ↓—decrease level.

The gut microbiota have a profound impact on host health. Intestinal bacteria perform numerous functions within the human body. These include aiding the digestive system, synthesising vitamins, stimulating the immune system, interacting with the intestinal epithelium, and influencing the host’s psychological well-being. Intestinal bacteria also play a key role in breaking down complex carbohydrates and fatty acids, producing SCFAs. An imbalance in gut microbiome composition can lead to various diseases, including allergies, type I diabetes, inflammatory bowel disease, necrotising enterocolitis and obesity. Maintaining a balanced microbiota, such as appropriate proportions of bacteria from the *Firmicutes* and *Bacteroidetes* phyla and sufficient numbers of probiotic bacteria, supports proper physiological functioning, potentially preventing or even alleviating certain diseases such as hypertension and hyperlipidaemia. The chronic use of specific medications, such as metformin, statins, proton pump inhibitors and antihypertensive drugs, can disturb the gut microbiota, leading to dysbiosis. Therefore, the administration of appropriate probiotic microorganisms into the body is crucial for preventing side effects such as diarrhoea, abdominal pain, nausea and bloating (Table 1 and Figure 2). Probiotics may also help prevent the onset of certain diseases by stabilising thyroid hormone levels, reducing total cholesterol and LDL-C levels in the blood, or lowering both systolic and diastolic blood pressure. Moreover, the gut microbiota influences the therapeutic effects of medications by affecting their absorption, metabolism and pharmacokinetics. An awareness of the intestinal interactions during medication use and understanding how to prevent dysbiosis is vital. Maintaining a healthy lifestyle, regular physical activity and a well-balanced diet are essential in supporting gut health.

## 4. Perspectives

Understanding the reciprocal relationship between pharmacotherapy and the gut microbiota is becoming crucial for the development of personalised medicine. Pharmacomicrobiomics—a science field that explores how the composition of the microbiota affects drug metabolism and efficacy, and how drugs, in turn, alter the microbiota—is playing an increasingly important role. There is growing interest in microbiota-targeted therapies, including the use of next-generation probiotics, synbiotics, and potentially genetically modified strains.

Advanced multi-omics technologies (metagenomics, transcriptomics, metabolomics), combined with artificial intelligence and machine learning, may allow for the prediction of patient responses to drugs based on their microbiota profile. Future clinical studies should also focus on the long-term impact of probiotic supplementation in the context of chronic pharmacotherapy, especially in metabolic, autoimmune, and cardiovascular diseases. Integrating microbiota knowledge into clinical practice may significantly enhance treatment effectiveness, reduce adverse effects, and support the body’s homeostasis.

## 5. Conclusions

The interaction between pharmacotherapy and the gut microbiota is increasingly recognised as a bidirectional and clinically relevant phenomenon. Commonly prescribed drugs, including antidiabetic agents, thyroid hormone replacements, statins, antihypertensive medications, and proton pump inhibitors, have been shown to modulate intestinal microbial diversity, abundance, and functionality. These alterations may influence not only therapeutic efficacy but also the occurrence of adverse effects, contributing to gastrointestinal intolerance, metabolic disturbances, or impaired drug absorption. Conversely, the composition and metabolic activity of the microbiota can affect drug bioavailability, pharmacokinetics, and treatment outcomes. The use of probiotics as a supplement to pharmacotherapy for chronic diseases may bring significant economic and clinical benefits by improving treatment efficacy, reducing adverse effects, and preventing dysbiosis. They are an important complement to therapy but do not replace medications. Further research is necessary to optimise and personalise this therapeutic approach. Emerging evidence highlights the potential of probiotics and synbiotics as adjuvant strategies to mitigate drug-induced dysbiosis, reduce gastrointestinal side effects, and improve clinical endpoints such as glycaemic control, lipid metabolism, thyroid hormone stability, and blood pressure regulation. However, current findings remain heterogeneous and strain-specific, underscoring the need for well-designed, large-scale clinical trials to establish causality and therapeutic guidelines. Integrating microbiota-targeted approaches into routine pharmacotherapy may represent a promising way to enhance efficacy, minimise adverse reactions, and support personalised medicine.

In summary, the integration of probiotics into pharmacological treatment supports gut health, enhances treatment outcomes, and may significantly reduce healthcare costs in the long term, offering multiple benefits for both patients and healthcare systems.

## Figures and Tables

**Table 1:** Effects of Selected Drugs on Gut Microbiota and Interactions with Probiotics. SCFA—short chain fatty acids; HbA1c—glycated haemoglobin; TC—total cholesterol; TG—triglycerides; LDL-C—low-density lipoprotein-cholesterol; SIBO—small intestinal bacterial overgrowth; TSH—thyroid-stimulating hormone; T3—triiodothyronine; -T4—thyroxine; PPI—proton pump inhibitor; CNS—central nervous system; ↑—increase level; ↓—decrease level.

Drug	Effect on Gut Microbiota	Interaction with Probiotics	References
Metformin	Microbiota changes: ↑ *Bacteroidetes*, ↑ *Actinobacteria*, ↑ *Proteobacteria;* ↓ *Firmicutes*, ↓ *Verrucomicrobia*; At genus level: ↑ *Bacteroides*, ↑ *Streptococcus*, ↑ *Collinsella*, ↑ *Escherichia*, ↑ *Clostridium*, ↑ *Subdoligranulum*,↑ *Akkermansia*, ↓ *Faecalibacterium*, ↓ *Ruminococcus*, ↓ *Roseburia;* ↑ SCFA-producing bacteria (*Butyrivibrio*, *Bifidobacterium*, *Megasphaera*, *Prevotella*, *Lactobacillus* spp);	Probiotics reduce gastrointestinal side effects and improve glycaemic (HbA1c, glucose) and insulin control;	[14,15,16,17,18,20,21,22]
Semaglutide	Microbiota changes: ↑ *Akkermansia*, ↑ *Alloprevotella*, ↑ *Alistipes*, ↑ *Muribaculaceae*, ↑ *Coriobacteriaceae*; ↓ *Romboutsia*, ↓ *Dubosiella*, ↓ *Lactobacillus*, ↓ *Ligilactobacillus*, ↓ *Enterorhabdus;*	No conclusive data on interactions with probiotics;	[25,26,27]
Levothyroxine	Microbiota changes: ↑ *Odoribacter*, ↑ *Enterococcus;* Dysbiosis observed in Hashimoto’s patients; ↓ *Lactobacillus*, ↓ *Bifidobacterium*; microbiota affects T3/T4 and micronutrient absorption;	Probiotics may stabilise TSH, improve thyroid function, and reduce drug dosage;	[31,32,34,35,38,39]
Atorvastatin	Microbiota changes: ↑ *Oscillibacter*, ↑ *Turicibacter*, ↑ *Anaerovorax*, ↑ *Parvibacter*, ↑ *Anaerotruncus*, ↑ *Bacteroides*, ↑ *Butyricimonas*, ↑ *Dorea*, ↑ *Mucispirillum*, ↓ *Parabacteroides*,↓ *Akkermansia*, ↓ *Rikenella*, ↓ *Christensensellaceae*, ↓ *Bifidobacterium*, ↓ *Tyzzerella*, ↓ *Lactobacillus;* intestinal barrier disruption; reduced effectiveness in poor microbiota;	Probiotics enhance effectiveness (↓ LDL-C, ↓ TC, ↓ TG); Changes in gut microbiota diversity: ↑ *Tenericutes*, ↑ *Bifidobacterium*, ↑ *Lactobacillus*, ↑ *Akkermansia*, ↓ *Proteobacteria*, ↓ *Escherichia*, ↓ *Eggerthella*, ↓ *Sutterella;*	[11,43,54,55]
Rosuvastatin	Microbiota changes: ↑ *Bacteroides*,↑ *Butyricimonas*, ↑ *Clostridium*, ↑ *Mucispirillum*, ↑ *Ruminococcaceae*, ↑ *Lachnospiraceae*; ↓ *Bacteroidetes;*	*L. acidophilus* enhances lipid-lowering effect; increased faecal levels in rats after combined therapy;	[11,46,52]
Amlodipine	Microbiota changes: ↑ *Akkermansia*, ↑ *Bacteroides*, ↑ *Lactobacillus*, ↑ *Ruminoclostridium*,↑ *Lachnospiraceae*, ↑ *Verrucomicrobia*	*L. plantarum* increases plasma drug levels—potential enhancement of absorption and bioavailability;	[71,72]
Captopril	Microbiota changes: ↑ *Tenericutes*, ↑ *Actinobacteria*, ↑ *Firmicutes*, ↓ *Bacteroidetes*, impacts gut permeability and CNS function;	No data on probiotic use with captopril;	[73,74]
Losartan	Restores F/B balance; Microbiota changes: ↑ *Verrucomicrobiaceae*, ↑ *Akkermansia*, ↑ *Pedobacter;* ↓ *Lactobacillaceae*; improves gut integrity and defensin production;	No specific data, but microbiota improvement may enhance therapeutic effect;	[75]
Proton Pump Inhibitors (PPIs: omeprazole, esomeprazole, etc.)	Microbiota changes: oral cavity: ↑ *Fusobacteriaceae*, ↑ *Leptotrichiaceae*, ↓ *Neisseriaceae*, ↓ *Veillonellaceae;* oesophageal: ↓ *Comamonadaceae*, ↑ *Clostridiaceae*, ↑ *Lachnospiraceae*, ↑ *Micrococaceae*, ↑ *Actinomycetaceae*, ↑ *Lactobacillales*, ↑ *Gemellales;* gastric: ↓ *Moraxellaceae*, ↓ *Flavobacteriaceae*, ↓ *Comamonadaceae*, ↓ *Methylobacteriaceae*, ↑ *Erysipelotrichaceae*, ↑ *Clostridiales;* stomach: ↑ *Streptococcaceae*, ↓ *Prevotellaceae*; small intestine: ↑ *Streptococcaceae*, ↑ *Staphylococcaceae*, ↑ *Enterobacteriaceae*, ↑ *Bacteroidaceae*, ↑ *Lactobacillaceae*, ↑ *Veillonellaceae*, ↑ *Clostridiaceae*, ↓ *Bifidobacteriaceae*; colon: ↑ *Enterobacteriaceae*, ↑ *Enterococcaceae*, ↑ *Lactobacillaceae*, ↓ *Ruminococcaceae*, ↓ *Bifidobacteriaceae;*	Decreases SIBO prevalence, reduces pathogenic bacteria, improves gut barrier function, and alleviates gastrointestinal side effects of long-term PPI use;	[87,89,91,92,93,94,95,96]

## Data Availability

No new data were created or analyzed in this study. Data sharing is not applicable.
